# The Impact of Video-Mediated Communication on Closed Wound Assessments in Postoperative Consultations: Conversation Analytical Study

**DOI:** 10.2196/17791

**Published:** 2020-05-05

**Authors:** Wyke J P Stommel, Harry van Goor, Martijn W J Stommel

**Affiliations:** 1 Centre for Language Studies Radboud University Nijmegen Netherlands; 2 Department of Surgery Radboud University Medical Center Nijmegen Netherlands

**Keywords:** video consultation, remote consultation, physical examination, assessment, patient-physician relationship, conversation analysis

## Abstract

**Background:**

Research on the use of video-mediated technology for medical consultations is increasing rapidly. Most research in this area is based on questionnaires and focuses on long-term conditions. The few studies that have focused on physical examinations in video consultations indicated that it poses challenges for the participants. The specific activity of wound assessment through video in postsurgery consultations has not yet been studied. Furthermore, a comparative analysis of face-to-face and video settings on the moment-to-moment organization of such an activity is original.

**Objective:**

The aim of this study was to examine the impact of video technology on the procedure of postsurgery wound assessment and its limits.

**Methods:**

We recorded 22 postoperative video consultations and 17 postoperative face-to-face consultations. The primary purpose of the consultation was to inform the patient about the final pathology results of the resected specimen, and the secondary purpose was to check on the patient’s recovery, including an assessment of the closed wound. The recordings were transcribed in detail and analyzed using methods of conversation analysis.

**Results:**

The way that an assessment of the wound is established in video consultations differs from the procedure in face-to-face consultations. In the consultation room, wound assessments overwhelmingly (n=15/17) involve wound showings in the context of surgeons reporting their observations formatted with evidentials (“looks neat”) and subsequently assessing what these observations imply or what could be concluded from them. In contrast, wound assessments in video consultations do not tend to involve showing the wound (n=3/22) and, given the technological restrictions, do not involve palpation. Rather, the surgeon invites the patient to assess the wound, which opens up a sequence of patient and physician assessments where diagnostic criteria such as redness or swollenness are made explicit. In contrast to observations in regular consultations, these assessments are characterized by epistemic markers of uncertainty (“I think,” “sounds...good”) and evidentials are absent. Even in cases of a potential wound problem, the surgeon may rely on questioning the patient rather than requesting a showing.

**Conclusions:**

The impact of video technology on postoperative consultations is that a conclusive wound assessment is arrived at in a different way when compared to face-to-face consultations. In video consultations, physicians enquire and patients provide their own observations, which serve as the basis for the assessment. This means that, in video consultations, patients have a fundamentally different role. These talking-based assessments are effective unless, in cases of a potential problem, patient answers seem insufficient and a showing might be beneficial.

## Introduction

Video consultations are generally found promising for use in the medical domain, especially due to advantages such as remoteness, convenience for patients and informal caregivers, and reduced anxiety [1-4]. However, the implementation of video consultations into real-world settings is complex. Most research in this area focuses on long-term conditions and is based on questionnaires to elicit patient and clinician experiences, reporting both positive and negative experiences with video consultations. The experiences often seem to depend on the context (eg, a long-term condition in which the clinician and patient have a pre-existing relationship, and on whether both parties are confident in dealing with technical issues [5,6]). Adaptation to the context can be accomplished by involving the patient in the choice of consultation modality. In a comparative study on video versus face-to-face consultations in follow-up care after colorectal cancer surgery, video consultations based on patient preference were shown to be equivalent to face-to-face consultations in terms of patient satisfaction and perceived quality of care [7]. The type of patients that might be most suitable for video consultations is unclear, but it is recognized that patients’ reasons and ability to use video consultation may change over time and with experience [8]. One of the advantages of the video format is that it affords visual access, which at least in theory enables physicians to visually assess what patients show. Nevertheless, physical examination has been regarded as problematic in the video setting [9]. Patients’ self-examinations in front of the camera, such as measuring weight, blood pressure, heart rate and rhythm, and oxygen saturation, appeared to be challenging in various respects [10]. One of the challenges was that patients had to do a physical examination while simultaneously making it visible to the clinician. Hence, visual access may not be just an advantage; it can also create new problems.

Conversation analytical studies of medical video consultations are beginning to uncover microlevel dimensions and challenges of video-mediated consultations [10-12], sometimes explicitly in comparison with copresent consultations [13]. Pappas and Seale [11,12] analyzed medical video consultations with a primary care physician or nurse and a patient at one end of the connection and a consultant (a medical specialist) at the other. The professional who was with the patient and, therefore, had direct perceptual access to the patient’s body, assessed the patient’s foot and used the visual channel to demonstrate the assessment to the physician on the other end [12]. Seuren et al [10] identified various challenges of video consultations in secondary care related to instructions for patients to self-measure oxygen levels and manipulate the camera and the body to capture what should be viewed by the physician.

A key domain of interest to medical video consultations is the physical examination, which requires the physician’s visual access to the patient’s body. Visual access is an affordance [14] of video-mediated interactions, despite the “fractured ecologies” [15] of the patient and the physician inherent to the interaction. It has been found that, by doing physical examinations, the remote physician transposes observational authority to the patient’s site [12]. Relatedly, examination conducted by patients themselves may enhance their autonomy with regard to their own health [10]. Hence, physical examination in video consultations may have the potential to instigate a shift in the physician-patient relationship or, more broadly, in the way that medicine is practiced.

When physicians examine patients, they may communicate the findings of their observations to the patient [16-18]. Simultaneously with the act of examination, physicians may produce talk that is subordinated to the examination, which is called online commentary. There is usually no mutual gaze and no response from the patient due to a lack of shared access to the object of evaluation (eg, a physician inspecting a patient’s ear). Alternatively, patients may be invited to provide an initial self-assessment as long as they have access to the object of examination.

There are two primary formats for communication along with physical examination, namely, reports of observations and assessments of what is observed [16,19]. In cases of an *observation report* (eg, “I don’t see”), the conclusions such as “looks good” should be drawn by the patient. With *assessments* of what is observed (eg, “that looks good”), it is the physician who presents a conclusion. Essentially, the power of both formats lies in the physician’s epistemic “ecological advantage” [16] to be able to perceptually (seeing, hearing, feeling) assess the state of the patient’s body. The criteria or “codes” for the evaluation that are discursively constructed in the interaction serve as an apparatus of the physician’s professional vision [20]. The ecological advantage, thus, encompasses rights with regard to both the examination and constructing observation in certain assessment categories.

Assessments are evaluations of objects and events in talk-in-interaction [21]. Assessments can be elicited both by verbal actions (eg, questions, prior assessments) and by embodied conduct or experience (cf [21,22]). Goodwin and Goodwin [23] discern assessments on distinct levels of organization, with assessment activities as one such level. Assessment as an activity refers to multiple participants jointly producing an assessment in multiple turns, using intonation, overlap, intensifiers, nods, and other resources. Relevant to such assessment activities is that the participants have differential access to the assessable, which is reflected in their talk. For instance, saying “that sounds good” attends to the fact that the assessable was available through a coparticipant’s description [23]. Displaying agreement on or producing concurring assessments is important in assessment activities. The same speaker can repeat an assessment; although, subsequent assessments may display diminished participation and, thus, bring the activity to a close. Overall, an activity of assessment is a structure that participants collaboratively bring to a climax and then withdraw from.

A specific occasion for the occurrence of assessments are “informative showings” [24], which involves showing something “new” such as the current state of the wound and a recipient who is informed by the showing. In the medical context, a showing enables the physician’s professional vision [20] as a basis for assessment rather than that joint visual perception that is achieved by the showing (cf [25-27]).

The question this paper addresses is how assessments of a surgical closed wound are collaboratively produced in video consultations where the physician lacks direct perceptual access to the assessable, which is available in the face-to-face setting. This question provides insights to the ways that video technology as a mode of communication affects clinical practice.

## Methods

The data consist of 39 video recordings of follow-up consultations after abdominal cancer surgery, including 17 copresent consultations (average length 13 minutes and 40 seconds) and 22 video consultations (average length 12 minutes and 20 seconds). The data were collected in the context of a study comparing the conversational organization of video-mediated consultations with regular consultations at the outpatient clinic during the first postoperative consultation after discharge [13]. The first postoperative consultation was chosen because of the potentially considerable burden of a visit to the outpatient clinic, and thus, video consultations had a potential advantage in this phase. The inclusion criteria were patients ≥18 years of age who had received abdominal cancer surgery. The exclusion criteria were an inability to give informed consent and a lack of proficiency in Dutch. A total of 39 patients participated (21 female and 18 male) often accompanied by one or more family members and 3 male surgeons who were experienced in video consulting before the start of the study. The type and complexity of the surgery was comparable for all patients; although, some were laparoscopic operations, which involved three or four small incisional wounds for the trocars, rather than one large wound. At discharge, the patients were informed about the follow-up consultation scheduled approximately 2 weeks after the operation. The goal of the follow-up consultation was explained as discussing the final pathology results and checking on recovery. The results sometimes involved bad news but were mostly brought on as a confirmation of what was expected. The question about recovery, including the wound assessments, usually came as a second order of business for the consultation [13]. The patients were offered follow-up consultations through video or as a regular consultation at the hospital. After they chose either of these options, they were informed about the study and asked to participate. They all gave their consent; although, 1 patient requested at a later time for us to not use the recording or transcript in any scientific publication or presentation of the study.

A waiver for medical ethical approval was obtained from Radboud Medical Center Ethical Committee in June 2017. The data were collected in June-July 2017 and March-June 2018. Each consultation was recorded using two cameras, one directed mainly at the surgeon and one at the patient and those that accompanied them, either in the consultation room or on the surgeon’s desktop computer. The recordings, thus, reflect the real-life circumstances of the surgeon, who does not have access to whatever the patient sees or hears such as delays, perturbations, or sequential mismatches (cf [28]). The particulars of the ecological setup at patients’ homes may have influenced some practical choices, for instance readjusting their body to the screen to make them visible rather than turning the camera toward the spot, which is another way to show things in video communication [29]. For the hospital recordings, the surgeons sometimes turned away the camera or even turned it off during the physical examination for ethical reasons; these recordings were not excluded from the data set, although they were inevitably inapt for detailed analysis of the examination. The consultations were transcribed based on conversation analysis conventions [30,31] (see Multimedia Appendix 1), and all names were replaced by pseudonyms.

To juxtapose assessments in copresent and video-mediated consultations, we first identified all wound assessment activities in the data and whether it involved a showing or not. The next step was to examine each case microanalytically using multimodal conversation analysis [32,33]. These analyses were inherently comparative, resulting in an understanding of the methods used for doing wound assessments contingent on the medium. In the next section, we discuss five illustrative cases of wound assessments, two in the copresent setting and three in the video setting, that are representative of our findings.

## Results

### Wound Assessment

We found that in the copresent setting, wounds were generally assessed on the basis of a showing of the wound. On the contrary, in the video-mediated setting, showings were rare. Table 1 provides an overview of the occurrence of wound assessment, wound assessment including showing, and no wound assessment.

**Table 1:** Frequency of showing-based and talk-based wound assessment in copresent and video-mediated consultations.

Group	Showing-based wound assessment, n (%)	Talk-based wound assessment, n (%)	No wound assessment, n (%)
Copresent (n=17)	15 (88)	1 (6)	1 (6)
Video-mediated (n=22)	3 (17)	12 (51)	7 (32)
Total (N=39)	18 (46)	13 (33)	8 (21)

In the following section, we first analyzed the face-to-face default method and then the default video-mediated communication (VMC) method. We found no communicative differences between laparoscopic wounds and other wounds. The two assessment procedures were mostly initiated by the physician who enquires about how the wound is healing. We also discuss one video consultation where the wound assessment is initiated by a patient who reports a potential problem. This allows for an in-depth understanding of the intricacies of wound assessment through video.

### The Face-to-Face Default Method: Showing-Based Wound Assessments

**Figure 1:**
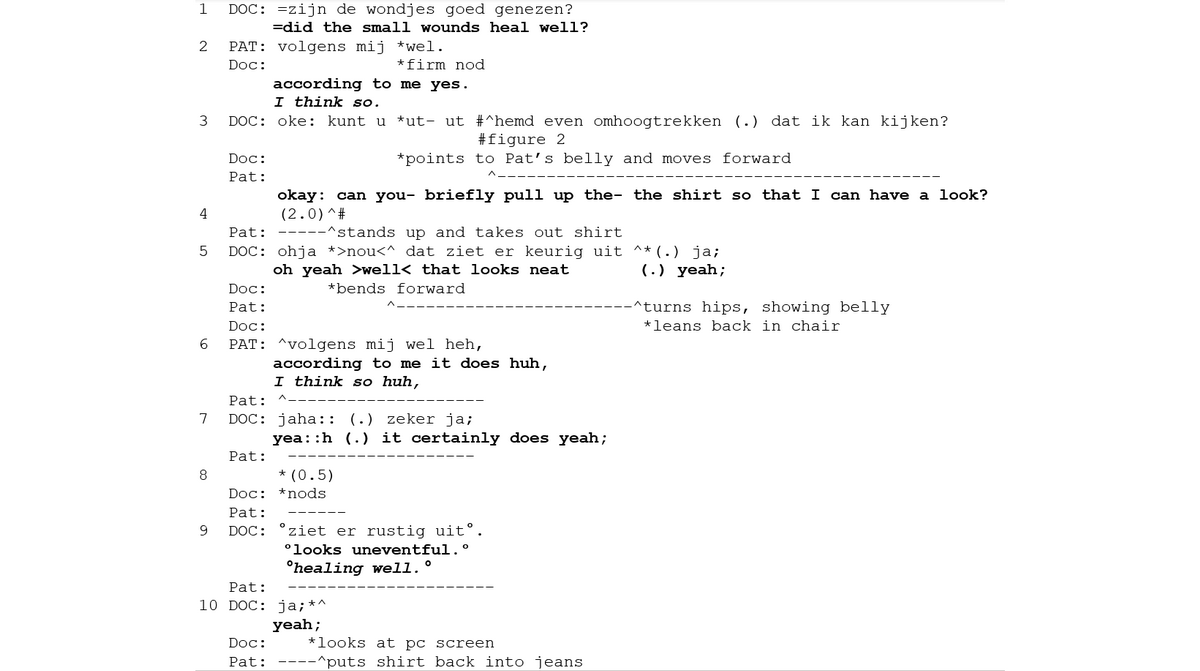
Excerpt 1. Regular consultation number 25 (video time: 5:50).

Showing-based wound assessments are characterized by a relatively stable structure. It is initiated by the physician asking whether the wound(s) are healing well. The patient’s answer is a tentatively positive evaluation of the wound, upon which the physician expands the sequence with a request to show the wound or an invitation to undress behind the curtain for a physical examination. During the showing or examination, the physician produces observation reports using evidentials and evaluations (“looks neat,” “looks uneventful”). The evaluations tend to be rather brief with general descriptors like “neat,” “good,” and “uneventful.” The excerpt in Figure 1 is an example of a copresent showing-based wound assessment. The physician is enquiring about the patient’s recovery, having asked about fever and illness (data not shown) and then about the wounds (line 1).

**Figure 2:**
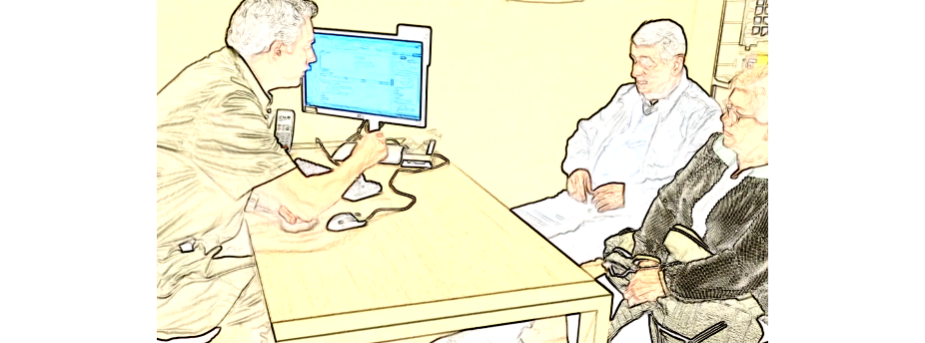
Screenshot for excerpt 1 (video time: 5:59).

The showing-based wound assessment activity begins with the physician’s yes/no-question (line 1), asking whether the small wounds healed well. The patient confirms with an epistemic downgrade (“according to me” line 2), thus, making an independent assessment by the surgeon a relevant next action. The request to show the wound “can you- briefly pull up the shirt” (line 3) displays a relatively high entitlement [34], thus, building on the structure of wound assessment as projected by the patient’s previous turn. The patient complies immediately (see line 3 and Figure 2), aligning with the activity. As soon as the wound is visible, the physician reports that it “looks neat” and confirms the patient’s initial evaluation (“yeah” line 5). Note that the physician uses an evidential (“looks”) to present a conclusion rather than an observation report, later rephrased as “looks uneventful” (line 9), which is produced softly and, thus, displays diminished participation and an orientation to activity closing [23].

**Figure 3:**
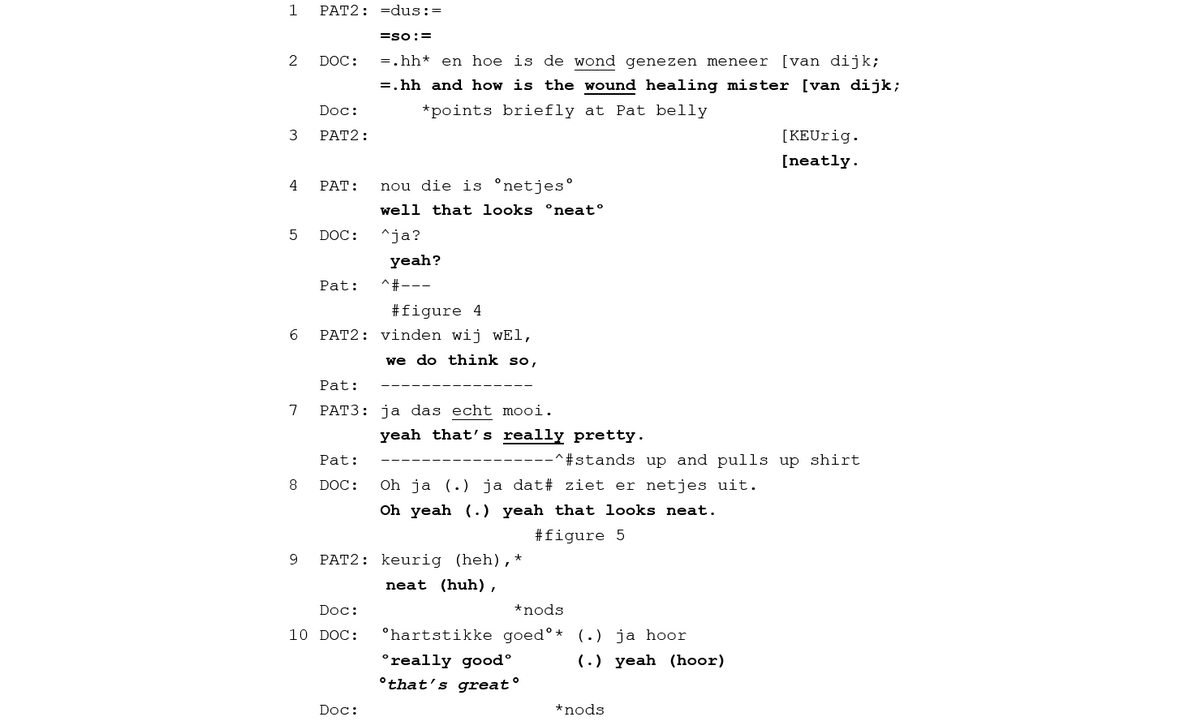
Excerpt 2. Regular consultation number 36 (video time: 7:37).

Giving visual access in the showing-based assessment activity does not need to be requested explicitly, as it was in Figure 1. The activity structure in the copresent setting allows for more subtle collaborative orientation to the relevance of showing as can be seen in Figure 3.

**Figure 4:**
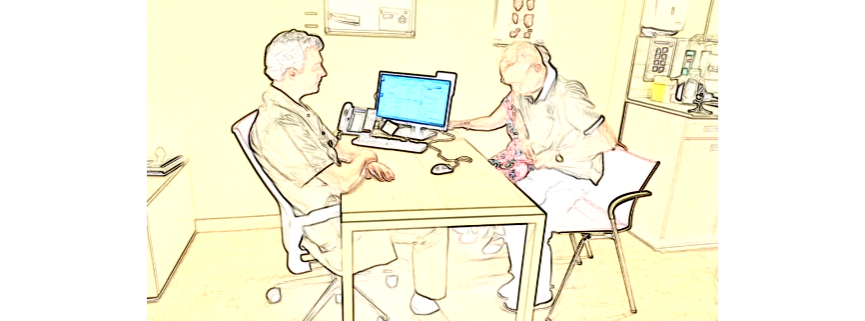
Screenshot 1 for excerpt 2 (video time: 7:38).

**Figure 5:**
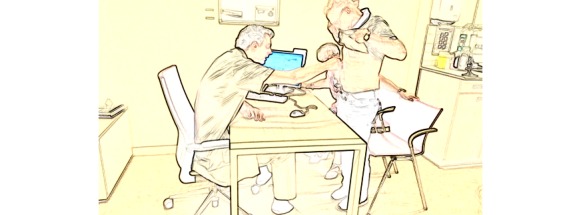
Screenshot 2 for excerpt 2 (video time: 7:41).

When asking how the wound is healing (line 2), the physician points at the patient’s belly, indirectly orienting to the show-ability of the wound. After a single-item positive assessment by the patient’s partner (line 3) and one from the patient (line 4), the patient rises to initiate a showing (Figure 4). Hence, he expands the initial positive assessments allowing the physician to independently self-assess the wound and arrive at a concurring assessment. That is, the patient’s claim about the showable wound makes the showing relevant as a way of facilitating independent access (cf [24]). As the showing is emerging nonverbally, the physician produces a checking question (“yeah?” line 5), which elicits an epistemically downgraded assessment from the patient’s partner (“we do think so” line 6) and an upgraded one from the patient’s daughter who sits off-camera (“really pretty” line 7). The physician then receives the now perceptually available wound as newsworthy (“oh” [35]; line 8), touches the belly just over the scar with two fingers (Figure 5), and assesses the wound using the same lexical form as the patient (“looks neat”). Hence, the structure of a wound-assessment activity in the consultation room is opened with a physician question and expanded with a showing, which leads to a concurring assessment by the physician. The physician assessment is formatted with an evidential (“looks”), displaying direct access to the assessable.

### The VMC Default Method: Talk-Based Wound Assessments

**Figure 6:**
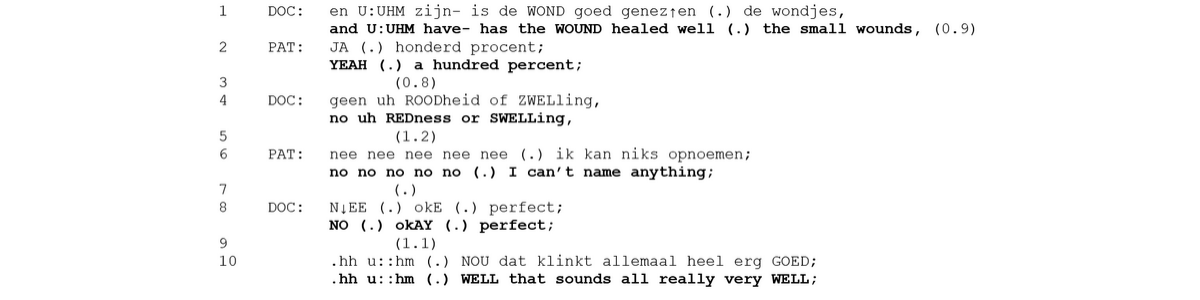
Excerpt 3. Video consultation number 16 (video time: 3:53).

The VMC default method is a talk-based assessment, which is characterized by a different structure and different epistemic marking compared to showing-based assessments. These sequences also typically begin with the physician asking whether the wound is healing well. Figure 6 is an example of a talk-based wound assessment typical for the video setting.

The assessment activity opening question received a positive assessment from the patient, in this case with the epistemic upgrade “a hundred percent” (line 2), which formulates the confirmation as an extreme case and, thus, legitimizes it [36]. The physician does not continue with a showing request nor does the patient initiate a showing. Rather, the physician poses a subsequent question to verify the patient’s answer. This question explicates two diagnostic criteria for wound assessments, namely, “redness” and “swollenness,” and makes relevant a confirmation of the absence of these symptoms from the patient. The patient then responds with multiple “no’s,” responding to not only the immediately preceding question but to the physician’s course of action, checking recovery [37]. This is elaborated with a more explicit assessment by the patient (“can’t name anything”), which again legitimizes the multiple sayings of “no” (cf [36]). The physician accepts and evaluates this answer (“perfect”), and then explicitly closes the “recovery” sequence with a qualified assessment: “.hh u::hm (.) WELL that sounds all really very WELL.” Note that this qualification displays the differential access [23] by the patient and physician to the wound (“sounds”), and acknowledges the patient’s evaluation(s) as the epistemic basis for this closing assessment. Hence, a talk-based wound assessment in VMC is an assessment activity similar to a showing-based assessment, but it involves questioning rather than showing. Furthermore, its climax assessment reflects differential access and is, thus, epistemically weaker than in a showing-based assessment.

**Figure 7:**
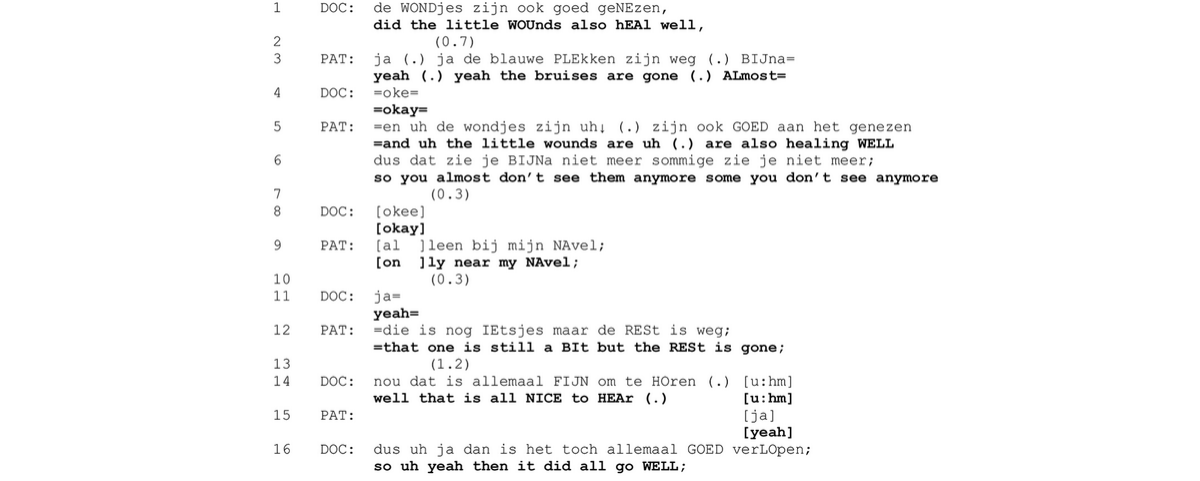
Excerpt 4. Video consultation number 12 (video time: 3:10).

Even when patients produce slightly less overtly rhetoric wound assessments than “hundred percent,” showings are not oriented to relevant next actions. This can be seen in Figure 7, in which the patient reports a potential minor problem with the wound (“only near my navel”).

In response to the activity-opening question (line 1), the patient first confirms, produces a general description of what can be seen on the body (“bruises are gone (.) almost”), and then gives an explicit verbal confirmation that the wounds are healing well. This is elaborated with a formulation of the visual observation of the wounds as the epistemic basis of this claim (“you almost don’t see them anymore some you can’t see anymore” line 6). Note that the patient uses the impersonal “you,” designing this claim as objective rather than as epistemically marked as her own observation.

The patient then expands the positive assessment with a minor problem (“only near my navel” line 9, “that one is still a bit” line 12) although this is contrasted with an overall positive assessment (“but the rest is gone” line 12), which proposes a closing of the assessment. The physician responds with the qualified assessment “well that is all NICE to Hear,” not orienting to the minor problem report but treating the patient’s wound assessment as relatively unspecific (“all”) and as news that he had no direct independent access to. The presented conclusion that follows (“so uh yeah then it did all go WELL”) is built on this general, positive news receipt and covers the whole surgery process, thus, moving out of the activity of wound assessment.

In summary, talk-based wound assessment sequences include the specification of diagnostic criteria (“redness,” “swollenness”), perceptual basis (“some you don’t see anymore”), or reference to a location on the body (“near my navel”). These may be elicited by the physician or volunteered by the patient. Generally, in such VMC talk-based assessments, physicians arrive at qualified wound assessments, marking them as epistemically grounded in the patient’s evaluation rather than in their own observation or examination.

### Patient-Initiated Wound Assessments in VMC

**Figure 8:**
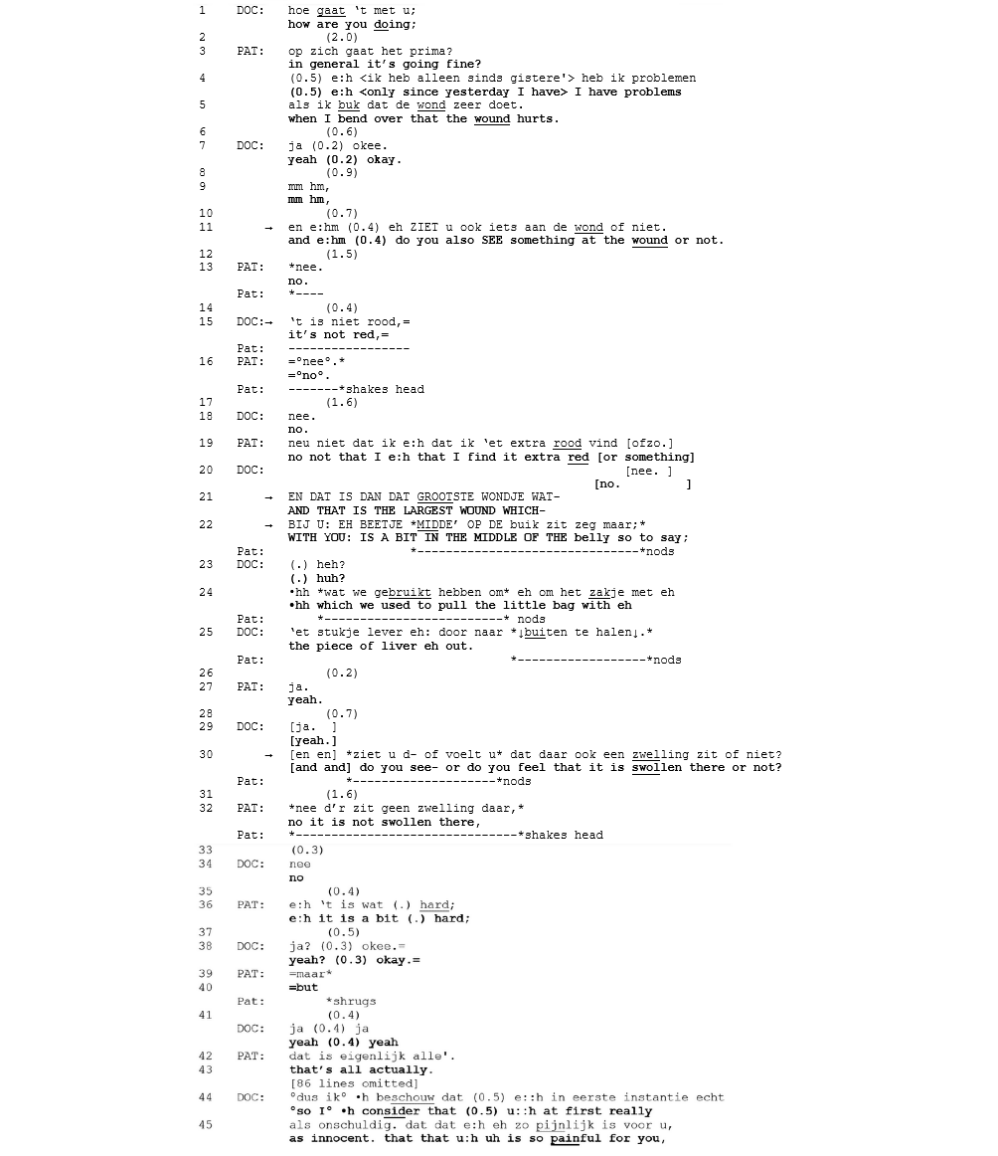
Excerpt 5. Video consultation 1 (video time: 1:54).

In the examples so far, the assessment sequences were initiated by the physician enquiring about the wound. However, wound assessments may also be initiated by patients rather than physicians with a report or question addressing some sort of trouble with regard to the wound. Although wound (or location) showings do occur (n=3/22 of video consultations), even patient-initiated sequences, which make a wound assessment relevant, may unfold as *talk-based* assessments in video consultations. In such cases, the interaction tends to be stretched over several sequences. The possibility of showing the wound is disregarded, despite the fact that visual access is available through the video connection. Figure 8 shows an example of a lengthy talk-based wound assessment in a video setting. In response to the question about how the patient is doing (line 1), she reports pain related to the wound as a first concern (cf [13]), which opens up the wound assessment activity.

To begin with, the patient refers to the viewable wound in her presentation of the problem (lines 4-5), which creates an opportunity for the physician to request a showing (a so-called “touched-off” showing [38]). However, rather than requesting that the patient show the wound, he accepts this initial problem account (“yeah (0.2) okay” line 7), which arguably projects history taking as a next activity [39]. Nevertheless, the physician does *not* take a turn, remains silent for 0.9 seconds, and then produces a continuer (“mhmm” line 9), creating a context for the patient to elaborate on the complaint. In the silence that follows (0.7 seconds), the patient does not continue, and the physician initiates the talk-based assessment activity. Hence, the initial problem report by the patient seems to create an interactional limbo in the structure of the assessment activity in which an opportunity to request a showing has passed.

The physician, asking whether the patient also sees something at the wound, orients to the viewability and, thus, potential show-ability of the wound. The patient denies something can be seen, and the physician expands by making explicit what could be seen, namely, redness (line 15). This further question also receives a “no,” but is then elaborated on with an account that implicitly proposes a scale of redness indicating the wound is not “extra red” (line 19). “Not extra red or so” implies the wound is (a bit) red, which may be a flag for trouble to the physician. Saliently, the patient produces this assessment without direct visual access (ie, she is not simultaneously inspecting the wound). Moreover, it is marked with an epistemic downgrade (“I find” line 19), thus, designing this observation as not only rooted in her earlier observation but also as subjective (or “subject-side” [40]). A showing could have resolved these issues, but this is not what happens. Rather, the physician checks whether the complaint is about the largest wound (line 21) and indicates roughly where this is located on the patient’s belly (line 22). In this way, again, a viewable (location on the belly) is talked about without being shown. After the participants have reached an agreement about which wound is being discussed (line 27 and 29), the physician launches another diagnostic question: “[and and] do you see- or do you feel that it is swollen there or not?” The seeing as a source of observation is redirected to feeling regarding the diagnostic criterion of “swollenness” (line 30). Hence, the patient is not invited to look “through the doctor’s eyes,” but to touch “on behalf of the physician.” This implies showing is now less relevant, as a showing might be perceptually inadequate to assess swollenness.

From this point onward, the patient reports tactile observations including that it is “not swollen there” (line 32), followed by a further potentially troublesome description “it’s a bit (.) hard” (line 36), which introduces yet another category. Nevertheless, the sequence is collaboratively closed with an orientation to the problem as minimal (“that’s all actually” line 43) and, thus, not in need of further discussion. The physician then starts a new but related sequence on the patient’s activities during the past weeks (data not shown), which eventually leads to his wound and pain assessment as “innocent” (line 45) with multiple disfluencies and hedges (“uh’s,” “at first really”), and an epistemic downgrade (“I consider that”). Hence, a talk-based assessment in cases of potential trouble may reside in talk to avoid a showing request. It includes the explication of multiple diagnostic criteria and may involve reference to various sensorial observations by the patient, and it eventually leads to a qualified wound assessment.

## Discussion

### Principal Results

Our primary finding is that video consultations differ from copresent consultations with regard to wound assessment. Talk-based wound assessment is the dominant trajectory in video consultations, while showing-based wound assessment is the dominant method in copresent consultations. Both trajectories are generally initiated with an informing question by the physician, but the subsequent steps differ. The activity continues with either a showing or examination of the wound, or with one or more questions enquiring the absence of specific diagnostic criteria (eg, redness, swollenness). Showing-based assessments work toward evidentially grounded general assessments (“neat,” “good,” “uneventful”), while talk-based assessments arrive at qualified assessments, which display a lack of direct access to the assessable (“sounds,” “I consider that”). Hence, wound assessments in video consultations are grounded in patient assessments, which implies a shift in clinical practice from primacy of the doctor’s gaze to the patient’s evaluation of how the wound(s) are healing. Even in cases of potential wound trouble in video consultations, physicians may rely on talk and avoid requesting a showing of the wound despite its apparent relevance. Such talk-based assessment sequences can be stretched substantially, with physicians bringing up multiple questions to enquire about symptoms and observations from the patient, both visual and tactile. Hence, despite the possibility of visual access and the interactional relevance, the participants displayed an orientation to avoid a showing in video consultations.

### Comparison With Prior Work

We may speculate about the reasons for the avoidance of showing closed surgical wounds in video consultations. A general reason could be that asking a patient to undress or show part of the nude belly or torso while being in the private sphere (usually the living room) with others potentially present and showing part of the nude body on camera are delicate things to do. In contrast, the hospital’s consultation room is marked with a clinical setup and assets (eg, physician wearing white coat, curtain, examination table, medical instruments), creating a context where showing the body and physical examinations may become relevant or may be expected by patients or physicians. Possibly, as participants’ experience with video interactions evolve, showing practices may occur more naturally. The avoidance of showings and, thus, direct visual access by the physician in video consultations implies that the “ecological advantage” [16] of physical examination may not or does not naturally apply to the video setting.

This means that physicians have less authority in diagnosing the wound and that patients are instead more agentive and epistemically amplified compared to face-to-face consultations. Similarly, Seuren et al [10] suggested that physical examination in video consultations may enhance patient autonomy, as patients become more active participants in the examination, having to handle instruments (eg, to measure oxygen in blood) or modify the camera. Nevertheless, physicians’ qualified assessments indicate a degree of uncertainty as a result of the restrictions of the medical armentarium inherent to the medium (eg, the impossibility of palpation) and reliance on patient reports and observations (cf [12]). This might explain why prior studies on physicians’ perspectives on applicability of video consultations revealed an anticipated need for physical examination as the main reason for not opting for video consultations [1,6]. The question is, however, under which conditions is it necessary to conduct a physical examination. In the majority of cases, talk-based assessment was sufficient to assess wound-healing, which implies the early postoperative phase is a context in which video consultations appear effective.

### Limitations

A limitation of our study is that we cannot exclude that the patients who chose a hospital consultation were more insecure about their recovery, including the wound(s), than those who opted for a video consultation. In that case, our findings could not only be explained by the medium of communication. However, in examining the data, we found multiple cases of patients in the hospital setting who did not present any insecurity with regard to their recovery, and we also found cases of potential insecurity (ie, patient reporting pain) in the video data. Another limitation is that the observed phenomenon may be related to the specific goal of the consultation. In our data, the reason for the consultation was the news delivery of the pathology results, and an examination of the wound was not explicitly announced. However, in the face-to-face consultations, showings and the physicians’ invitations to “have a look” were utterly unproblematic. Nevertheless, it is possible that in video consultations where the goal of the interaction is more closely linked to examination, medically relevant showings are more common and are also volunteered by patients (cf [10,12]). Patients may even close the curtains or do the video consultation from their bedroom. Hence, medical assessment practices are likely to further evolve with participants’ growing familiarity with video-mediated interactions.

### Conclusions

Overall, it has become clear that video-mediated and copresent medical interactions differ with regard to assessments of medical assessables such as wounds. It was particularly the comparative perspective that yielded new insights, providing evidence of normative orientations with regard to showing that intersect the medical dimension of the talk and the medium of communication. This underscores the relevance of the communication channel for the organization of institutional talk-in-interaction [41] and shows that juxtaposing equivalent interactions through different media is worthwhile, particularly when the choice for the one or the other medium is an ”emic” choice for both the patient and—in a different way—the physician. New communicative affordances such as patients sharing images of their body parts with their phones are likely to further affect medical interactions.

A practical implication of our study is that physicians may have to do “extra work” in video consultations to facilitate showing-based assessments. Furthermore, they should consider under which circumstances (eg, closed wound inspection) a hospital visit is more suitable than a video consultation. Another practical implication is that talk-based assessment seems to reduce the physician’s medical authority, as it ascribes more authority to the patient. This reliance on patient observation and judgement is in line with increased self-management as a form of patient empowerment in video consultations [10,42], which is generally regarded as beneficial. It nevertheless seems important that practitioners are aware of potentially shifting authority.

## Appendix

Multimedia Appendix 1 Transcription conventions.

## Abbreviations

VMC: video-mediated communication
