# Supplementary material for: The Impact of Video-Mediated Communication on Closed Wound Assessments in Postoperative Consultations: Conversation Analytical Study
Source: J Med Internet Res. 2020 May 5;22(5):e17791. doi: 10.2196/17791 (PMC7238083; doi:10.2196/17791)
Supplement: Multimedia Appendix 1 [file jmir_v22i5e17791_app1.pdf]

Transcription conventions

Data are represented in two-line transcripts, occasionally three lines. The first represents the original utterance in Dutch; the second line gives the English translation word by word, staying as close as possible to the Dutch. Where relevant, a more understandable/idiomatic English translation is given in the third line, in italics.

[	A left bracket indicates the onset of overlapping talk.
]	A right bracket indicates the end of overlapping talk.
=	Equal signs ordinarily come in pairs and indicate that the second line was “latched” to the first.
(0.0)	Numbers in parentheses indicate silence represented in seconds.
(.)	A dot in parentheses indicates a “micropause” of less than 0.2 seconds.
::	Colons indicate prolongation of the immediately prior sound.
-	A hyphen indicates a cut-off.
hh	The letter “h” indicates audible outbreath. The number of “h”s represents the length of the outbreath.
.hh	The letter “h” preceded by a dot indicates audible inbreath. The number of “h”s represents the length of the inbreath.
yes	Underlining indicates emphasis.
YES	Upper case indicates that a word or utterance is markedly loud.
°yes°	A word or utterance enclosed by two degree signs is markedly quiet or soft.
.	A dot at the end of an utterance indicates strongly falling intonation.
;	A semicolon at the end of an utterance indicates falling intonation.
?	A question mark at the end of an utterance indicates strongly rising intonation.
,	A comma at the end of an utterance indicates slightly rising intonation.
*	Asterisks indicate onset and ending of bodily behaviors of physician during talk on a separate line.
^	Carats indicate onset and ending of bodily behaviors of patient during talk on a separate line.
#	Hashtag refers to an image (figure) as it is related to the talk.
